# Supplementary material for: CCN1 interacts with integrins to regulate intestinal stem cell proliferation and differentiation
Source: Nat Commun. 2022 Jun 3;13:3117. doi: 10.1038/s41467-022-30851-1 (PMC9166801; doi:10.1038/s41467-022-30851-1)
Supplement: Supplementary file 1 — Supplementary information [file 41467_2022_30851_MOESM1_ESM.pdf]

Supplementary information

CCN1 interacts with integrins to regulate intestinal stem cell proliferation and differentiation

Jong Hoon Won, Jacob S. Choi, and Joon-Il Jun

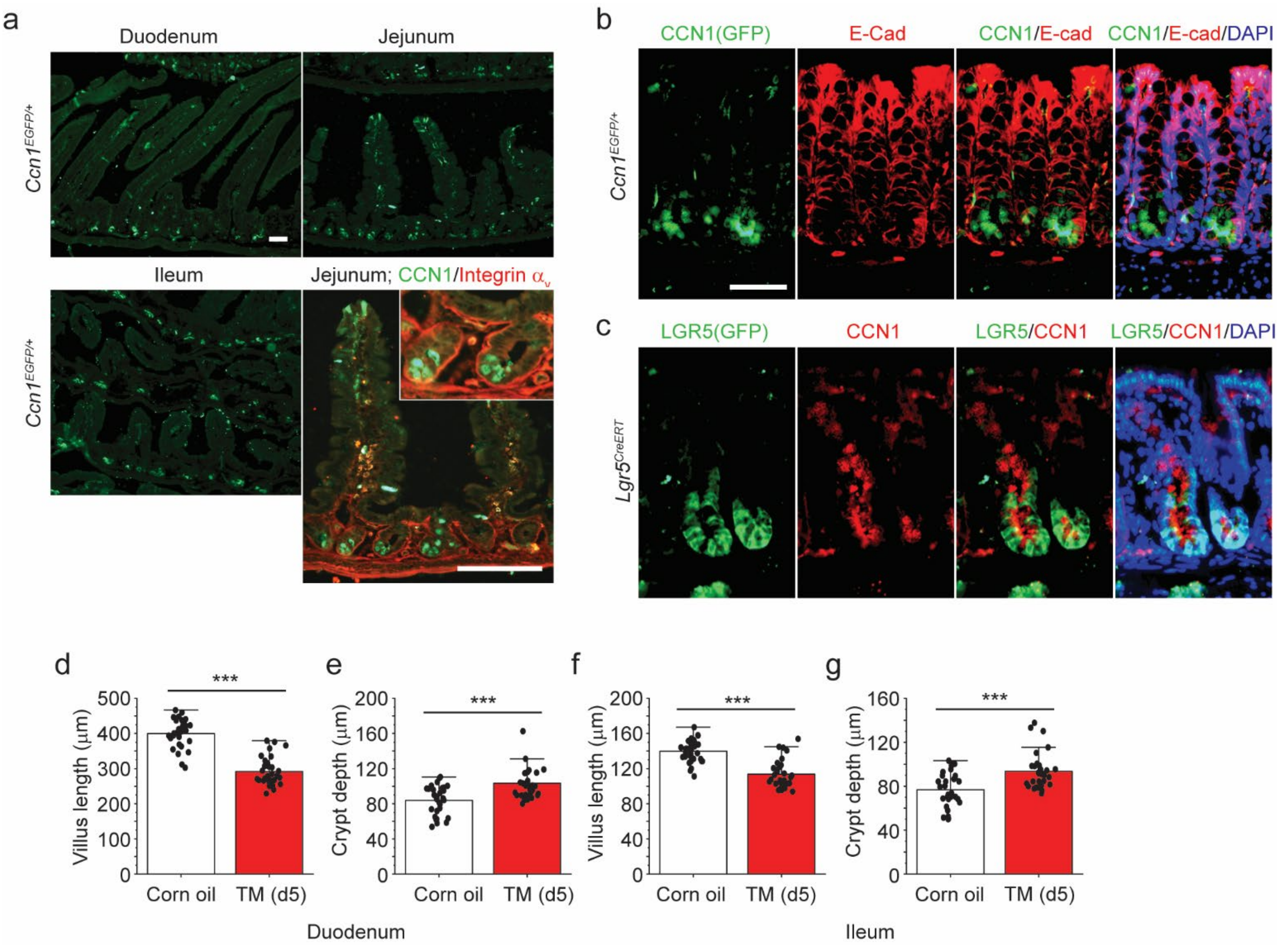

Supplementary Figure S1. Analysis of CCN1 expression in the small intestine and colon epithelium, and changes in crypt/villus length with *Ccn1* deletion in *Lgr5*+ ISCs. a. *Ccn1* expression (GFP) in the small intestine (duodenum, jejunum, and ileum) in *Ccn1*$^{EGFP/+}$ mice and immunofluorescence staining of integrin α_v in the jejunum section. The crypt area is enlarged, showing CCN1 (GFP, green) and integrin α_v (red). Bar: 100 μm. **b.** *Ccn1* expression (GFP) in colon epithelium in *Ccn1*$^{EGFP/+}$ mice. E-cadherin (E-Cad, red) stains epithelial cells. DAPI is counterstaining. **c.** Immunofluorescence staining of CCN1 (red) and GFP (*Lgr5*+ ISCs) in the colon of *Lgr5*^{CreERT} *mice*. Representative images from 3 independent stainings. Bar: 50 μm. **d-g.** The quantification of villus and crypt length in the duodenum (**d, e**) and ileum (**f**, **g**) of *Ccn1*^{DLgr5} mice with corn oil or TM for 5 days. The length was calculated from 6-10 randomly selected fields per section of H&E staining (*n*=5) using ImageJ. Data represent mean ± SEM. One-sided *t*-test. *** $p<0.001$. Source data and the exact *p*-values are provided in a Source Data file.

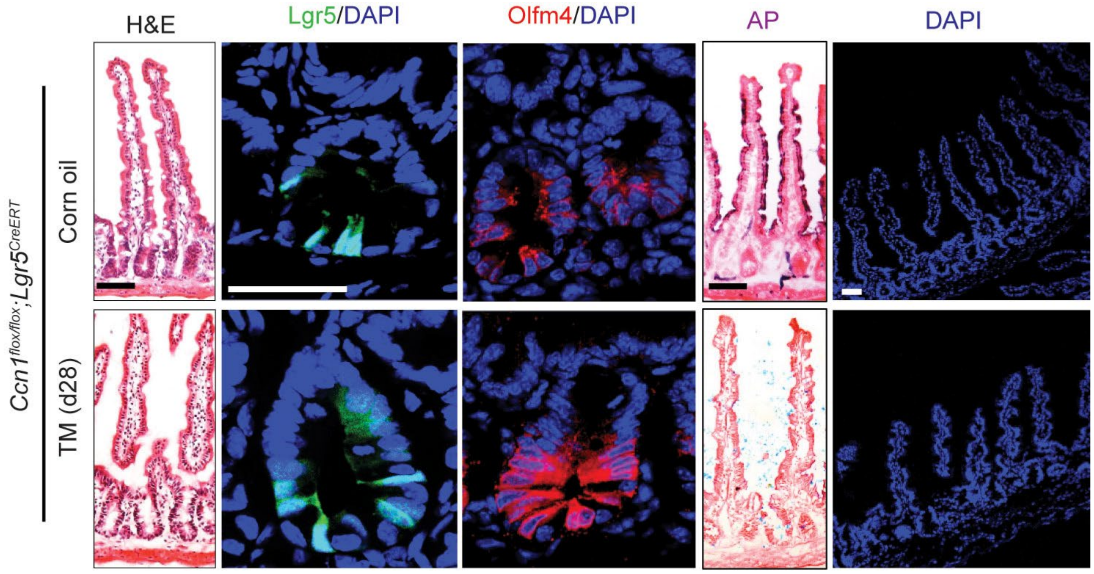

Supplementary Figure S2. Histological evaluation of the jejunum in *Ccn1$^{\Delta Lgr5}$* mice. The jejunum sections of *Ccn1$^{\Delta Lgr5}$* mice treated with corn oil or TM for 28 days were stained with H&E and visualized for *Lgr5*+ ISCs (GFP, green) and Olfm4 (red) using Immunofluorescence. AP staining is to mark enterocytes. DAPI is counterstaining to show the overall structure of the jejunum. Representative Images from 3 independent stainings. Bars: 50 μm.

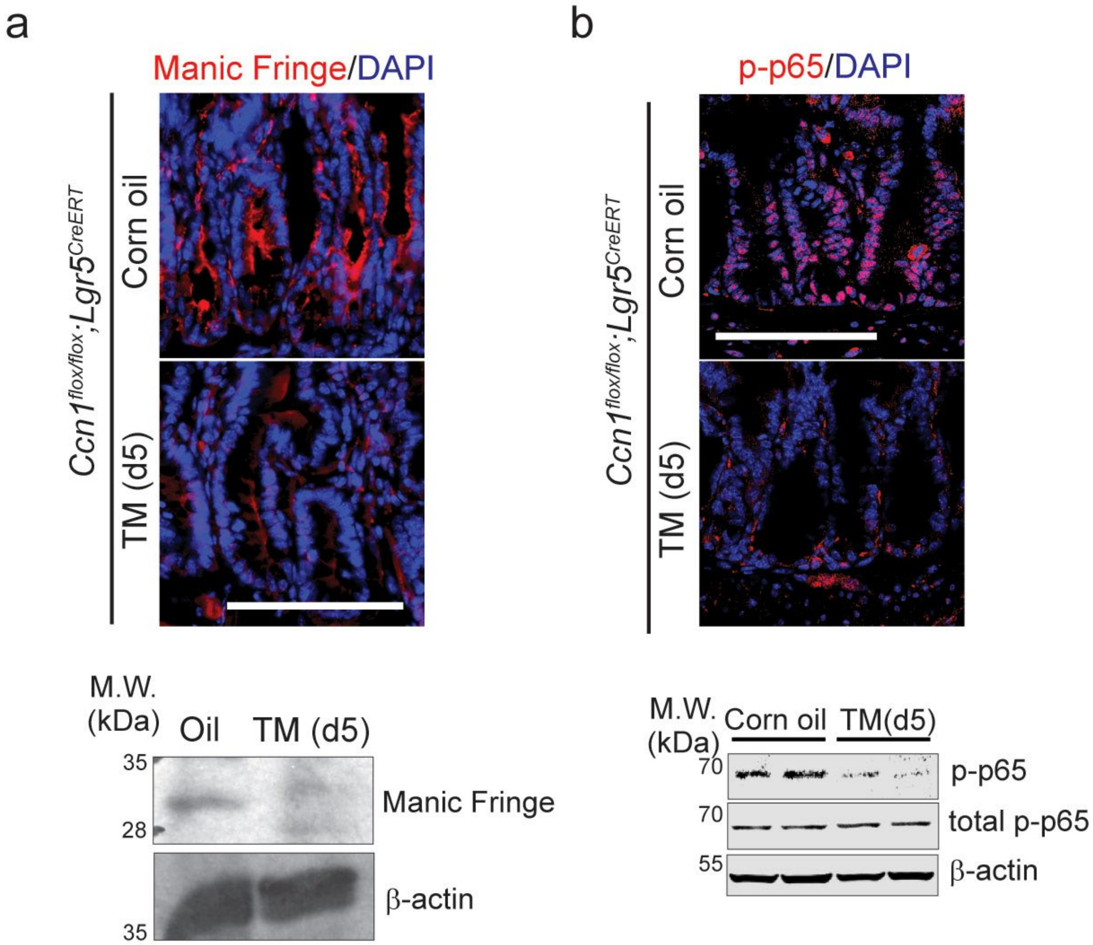

Supplementary Figure S3. Reduced MFNG expression and NF-κB activation in the crypt of mice with *Ccn1* deletion in *Lgr5*+ ISCs. a, b. The jejunum sections of *Ccn1*$^{\Delta Lgr5}$ mice treated with corn oil or TM for 5 days were analyzed for Manic Fringe (MFNG) and p-p65 in immunofluorescence staining (*top* panels) and immunoblot analysis (*bottom* panels). DAPI is counterstaining. β-actin as a loading control. Representative images from 3 independent replicates. Bar: 100 μm.

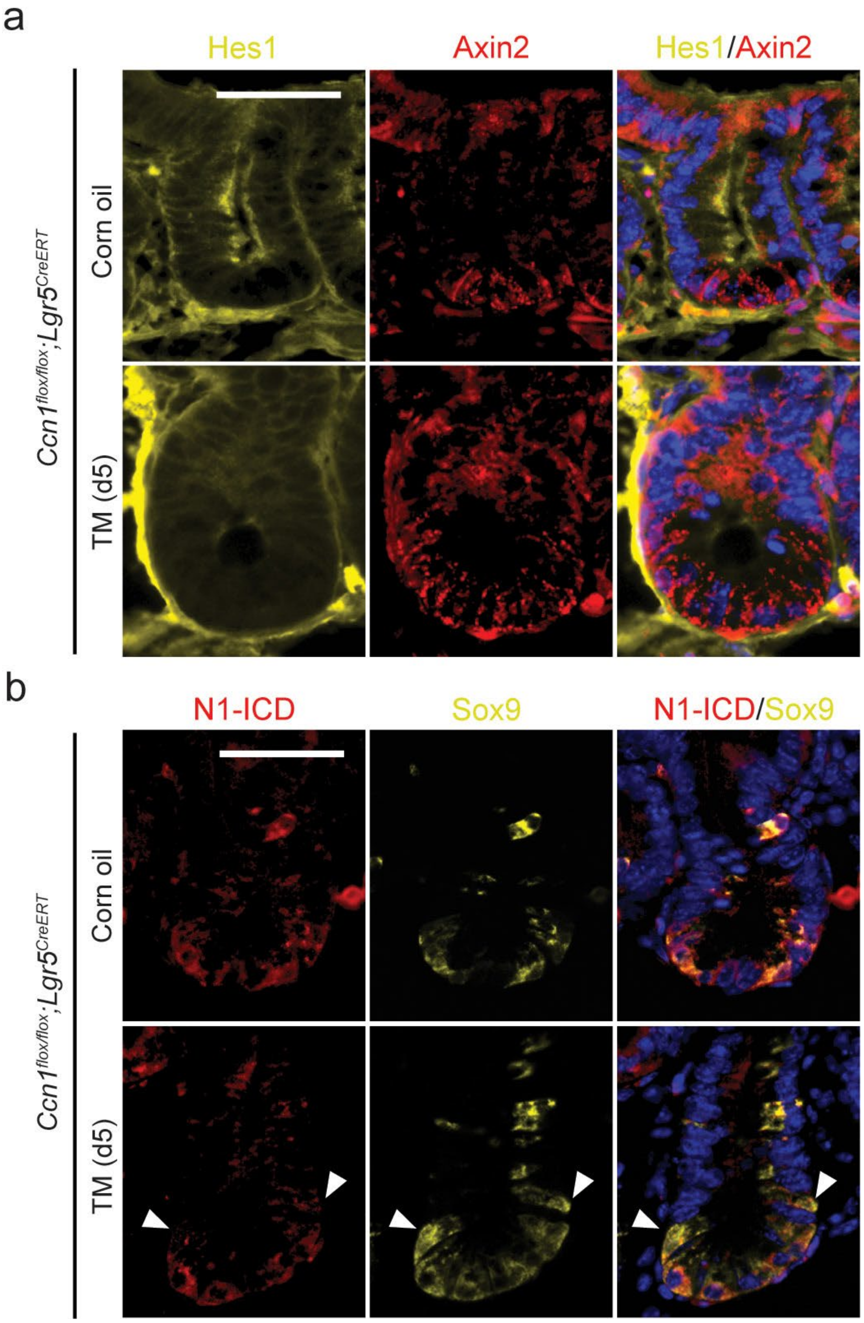

Supplementary Figure S4. Changes in Notch and Wnt signaling in the crypt of mice with *Ccn1* deletion in *Lgr5*+ ISCs. a, b. The jejunum sections of *Ccn1*$^{\Delta Lgr5}$ mice treated with corn oil or TM (*n*=3 each) for 5 days were analyzed for Hes1 (yellow) and Axin2 (red, **a**), and N1-ICD (red) and Sox9 (yellow, **b**) in double immunofluorescence staining. DAPI is counterstaining. Arrowheads point cells with increased Sox9 but much reduced N1-ICD at the border of the stem cell compartment and TA zone. Representative images from 4 independent stainings. Bar: 50 μm.

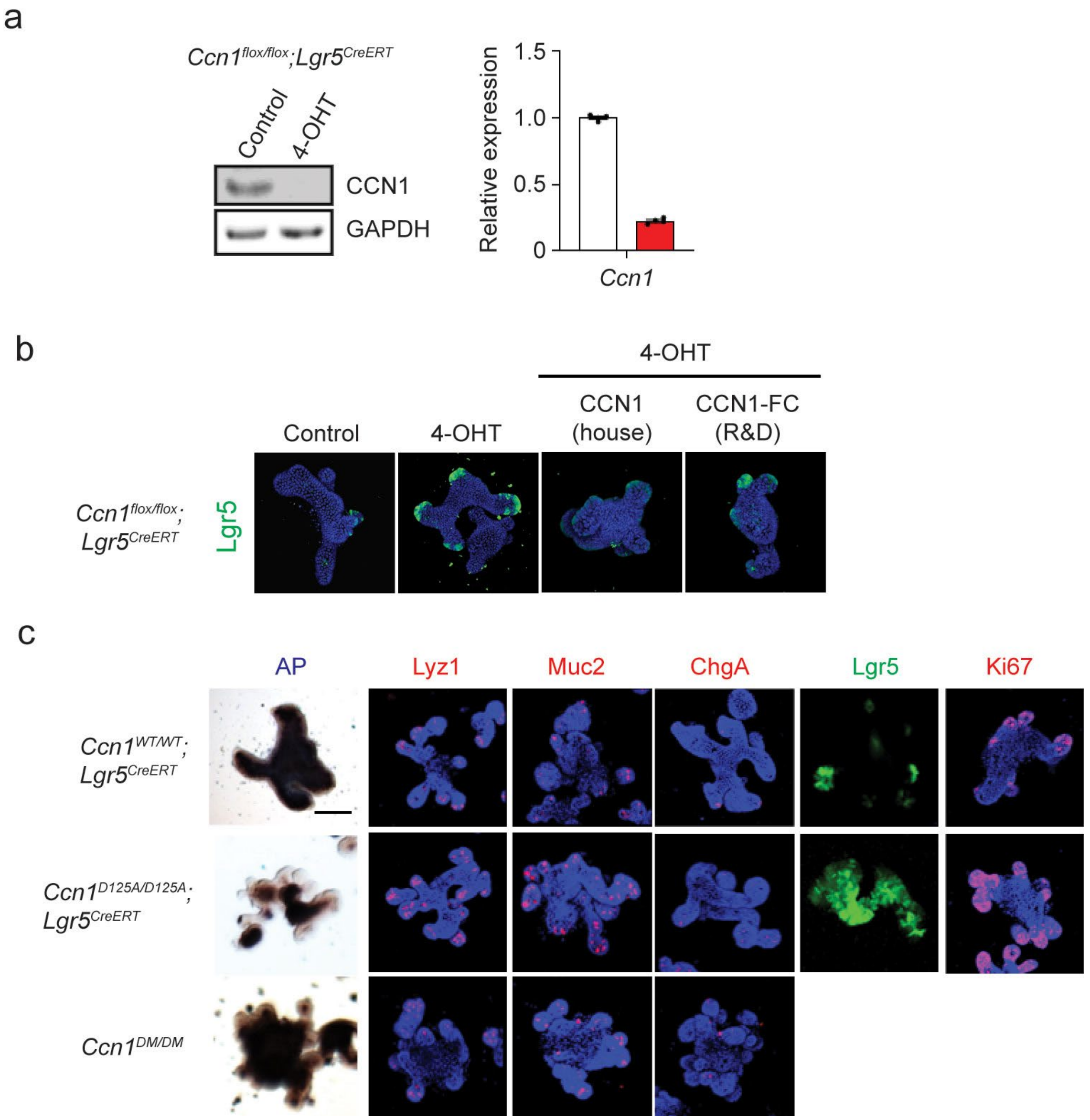

Supplementary Figure S5. Integrin-mediated CCN1 function in differentiation and proliferation of ISCs. a. Immunoblot and qPCR analysis to confirm *Ccn1* deletion in *Ccn1*$^{\Delta Lgr5}$ organoids with the treatment of 4-OHT (1 μM) for 5 days. Immunoblot images are representative of 3 replicates and qPCR data represent the mean ± SEM of 4 biological replicates. **b.** The effect of CCN1 add-back on the expansion of *Lgr5*+ ISCs. CCN1 proteins (4 μg per ml) from either house or R&D systems were tested for comparison. **c.** AP staining and immunofluorescence staining of markers (Lyz1, Muc2, ChgA, Ki67; red, *Lgr5*+GFP; green) in organoids from *Ccn1*$^{WT/WT}$*; Lgr5*^{CreERT}, *Ccn1*$^{D125A/D125A}$*; Lgr5*^{CreERT}*, or Ccn1*$^{DM/DM}$ mice. Representative images from 3 independent stainings. Bar: 100 μm. Source data are provided in a Source Data file.

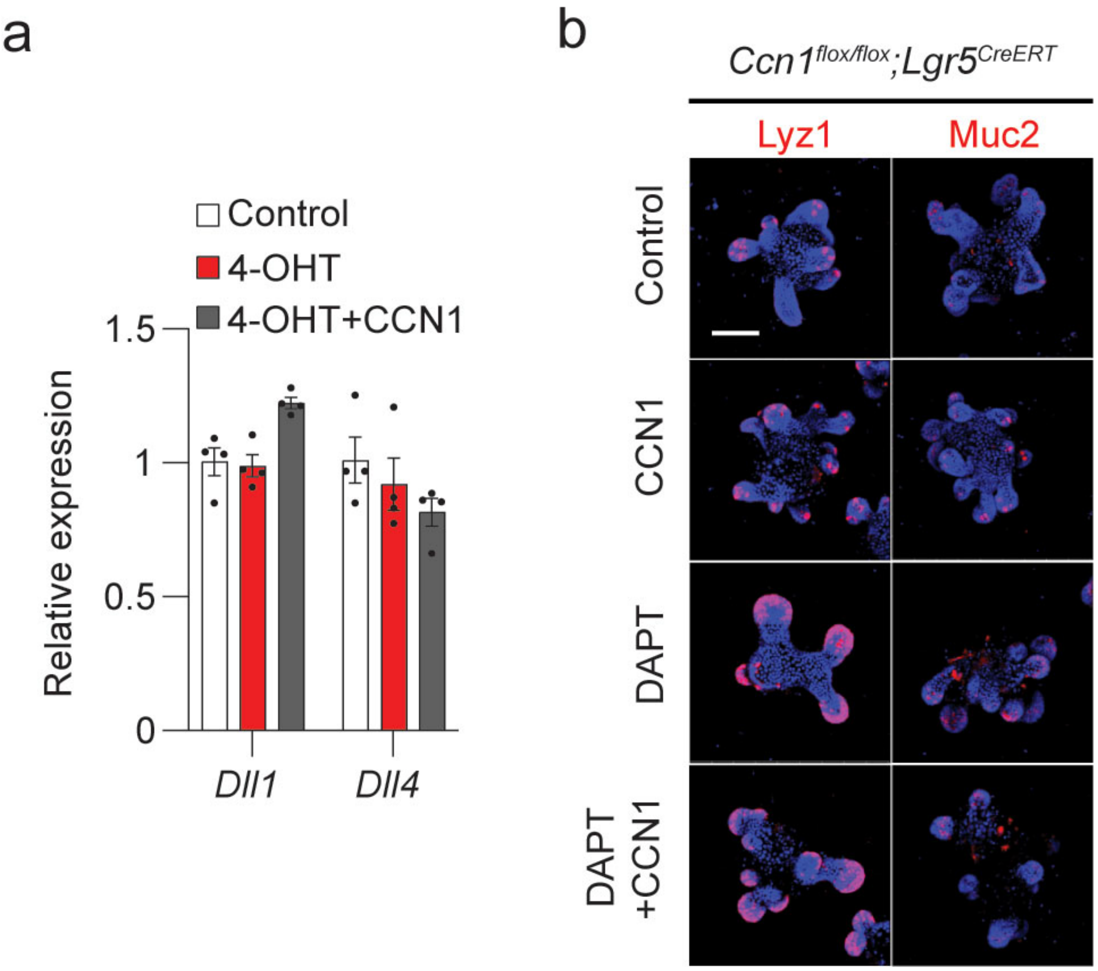

Supplementary Figure S6. Analysis of Notch ligands *Dll1* and *Dll4*, and DAPT effect on secretory cell differentiation. a. qPCR analysis of *Dll1* and *Dll4* expression in 4-OHT (1 μM)-treated *Ccn1*$^{\Delta Lgr5}$ organoids with or without CCN1 add-back (4 μg per ml). Data represent the mean ± SEM of 4 biological replicates. **b.** Immunofluorescence staining of secretory cell markers (Lyz1 and Muc2; red) in *Ccn1*$^{\Delta Lgr5}$ organoids treated with γ-secretase inhibitor DAPT (10 μM) in the presence or absence of CCN1 (4 μg per ml). DAPI is counterstaining. Bar: 100 μm. Images are representative of 3 independent stainings. Source data are provided in a Source Data file.

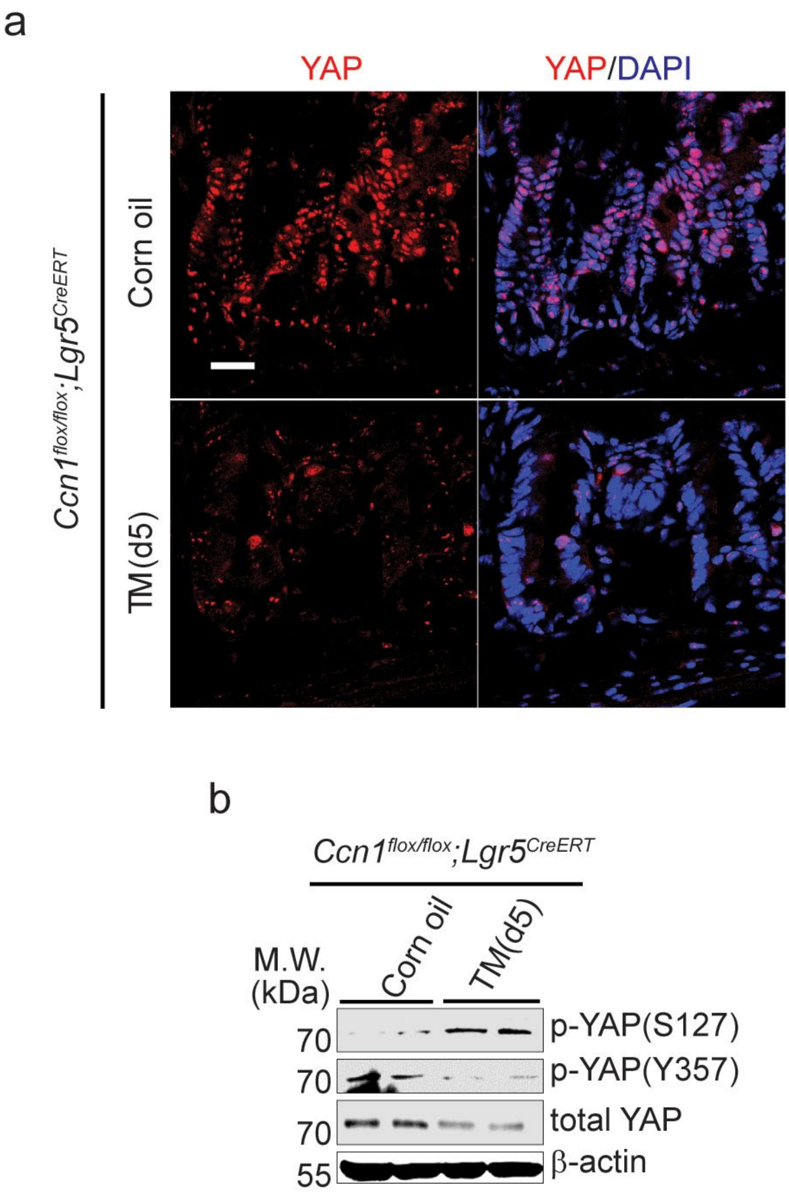

Supplementary Figure S7. Reduced YAP activation in the crypt of mice with *Ccn1* deletion in *Lgr5*+ ISCs. *Ccn1*$^{\Delta Lgr5}$ mice were treated with corn oil or TM for 5 days. **a.** Immunofluorescence staining of YAP (red) in the jejunum sections. Representative images from 3 independent stainings. Bars: 20 µm. **b.** Immunoblot analysis of YAP phosphorylation (S127 or Y357). β-actin as a loading control. Representative images from 2 biological replicates.

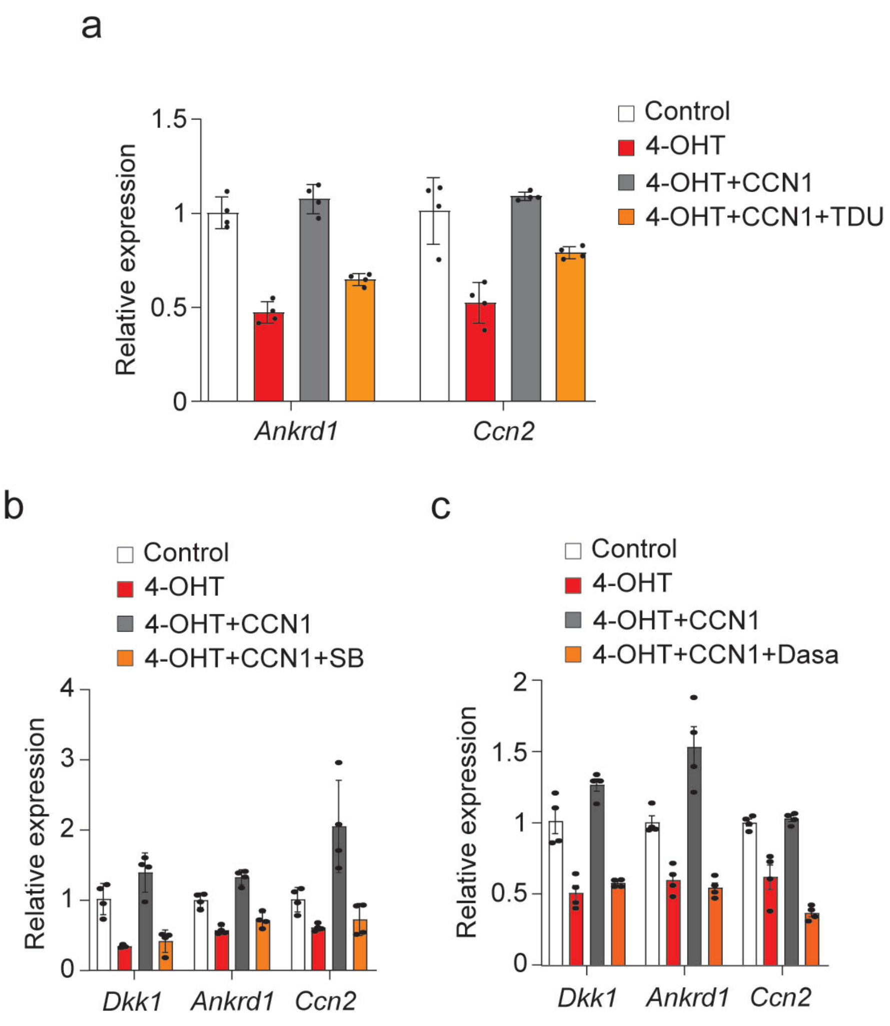

Supplementary Figure S8. CCN1 regulates *Dkk1* expression through the integrin α_v–Src-Yap axis. a-c. qPCR analysis of *Dkk1* and other YAP target genes (*Ankrd1 and Ccn2*) in *Ccn1*$^{\Delta Lgr5}$ organoids treated with vehicle or 4-OHT (1µM) for 5 days. CCN1 (4 µg per ml) was added to organoids 1 day after 4-OHT. Inhibitors used are Super-TDU (50 nM, **a**), SB273005 (1µM, **b**), and dasatinib (10 nM, **c**). Data represent the mean ± SEM of 4 biological replicates. Source data are provided in a Source Data file.

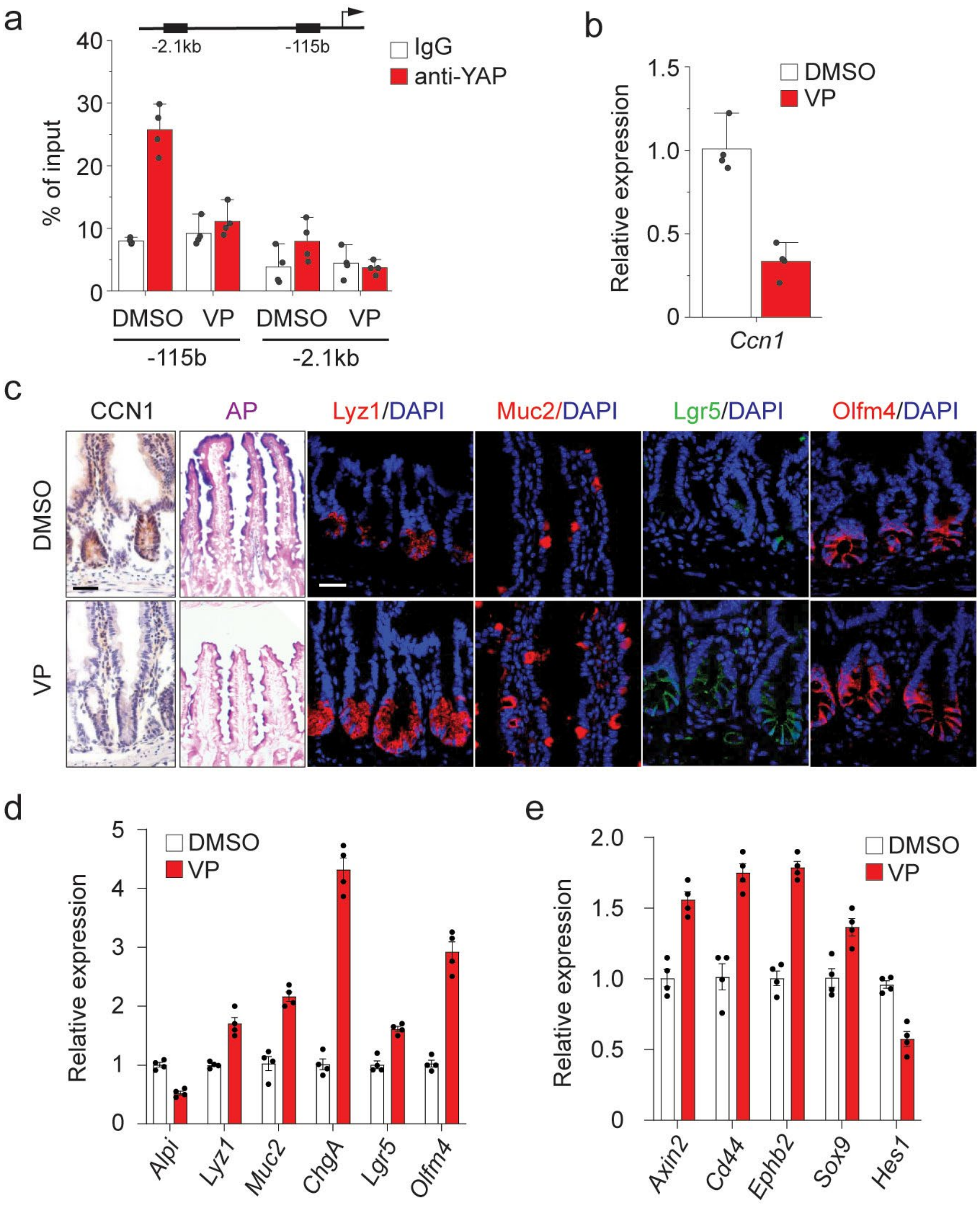

Supplementary Figure S9. YAP transcriptionally activates *Ccn1* and regulates ISC homeostasis. Control *Ccn1*$^{\Delta Lgr5}$ mice (*n*=3, each) were administered with either DMSO or verteporfin (VP, 30 mg per kg) for 5 days and intestinal tissues (jejunum) were harvested and processed for crypt isolation or immunohistochemistry. **a.** Chromatin immunoprecipitation (ChIP) assay of YAP binding on the *Ccn1* promoter in the mouse intestinal crypts. An anti-YAP antibody (2 μg) or isotype control IgG (rabbit) was used for IP. Primer sets target -115b or -2.1kb of the *Ccn1* promoter. Data are presented as a percentage of input (mean ± SEM of 4 biological replicates). **b.** qPCR analysis of *Ccn1* expression in the crypts. Data represent the mean ± SEM of 4 biological replicates. **c.** Immunohistochemistry of CCN1 and epithelial cell markers (*Lgr5*+GFP, Lyz1, and Muc2) and AP staining on the jejunum section. Representative images from 3 independent stainings. Bars: 20 μm. **d, e.** qPCR analysis of lineage markers (*Alpi, Lyz1, Muc2, ChgA, Lgr5, and Olfm4*; **d**) and Wnt target genes (*Axin2*, *Cd44*, *Ephb2*, and *Sox9*) and Notch target gene *Hes1* (**e**). Data represent the mean ± SEM of 4 biological replicates. Source data are provided in a Source Data file.

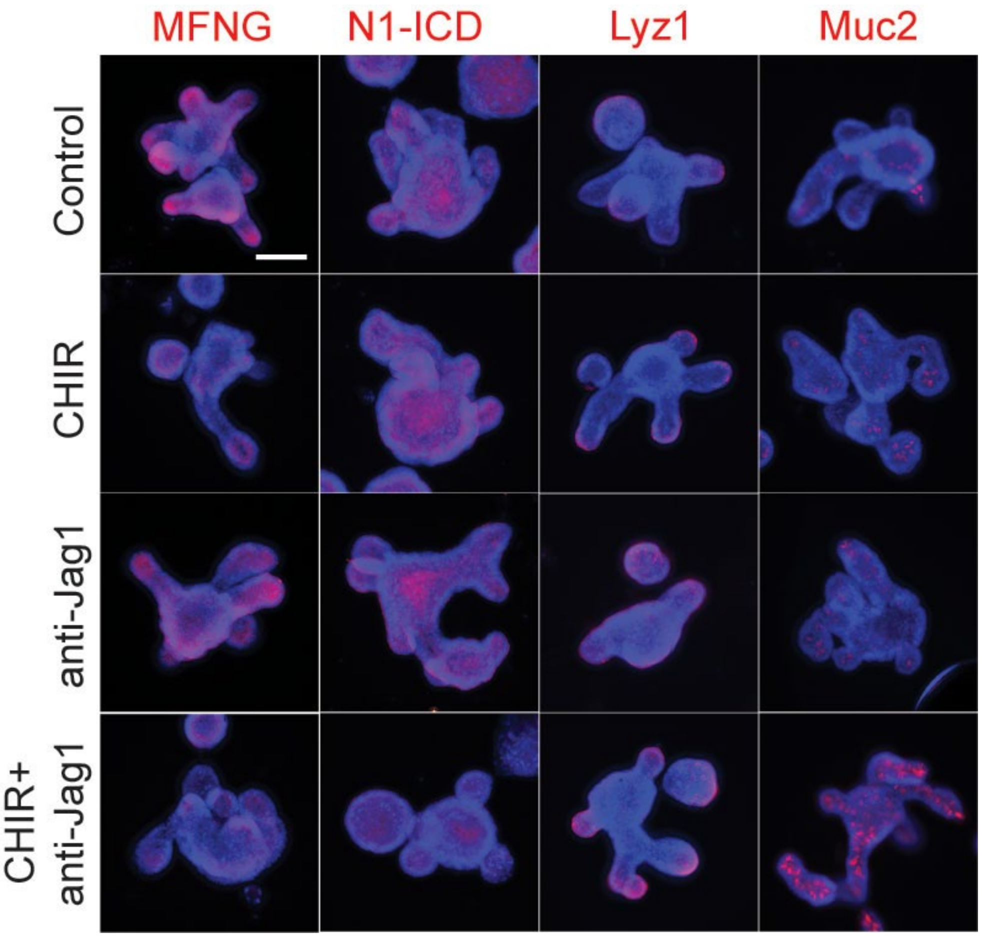

Supplementary Figure S10. Jag1 inhibition with Wnt activation resulted in secretory cell differentiation. Crypt organoids from control mice (C57BL/6) were treated with CHIR99021 (5 µg per ml), a neutralizing anti-Jag1 antibody (3 µg per ml), or both every 2 days for 6 days. Immunofluorescence staining of MFNG, N1-ICD, Muc2, and Lyz1. DAPI is counterstaining. Representative images from 3 independent stainings. Bar: 100 µm.

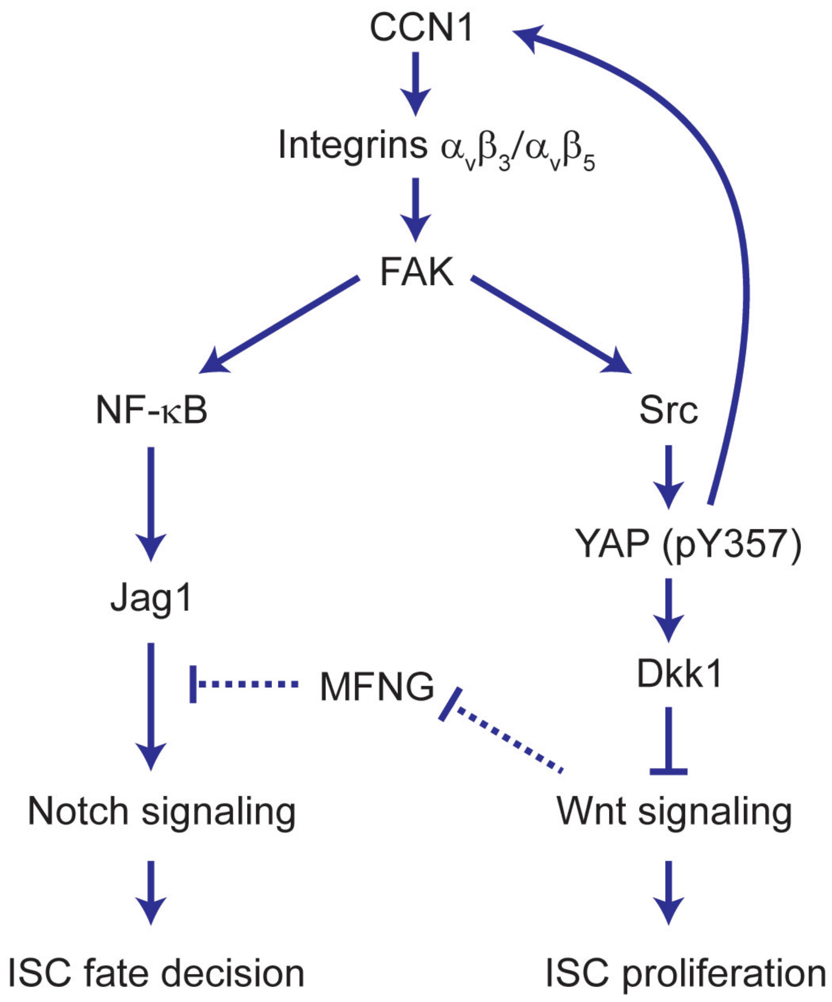

Supplementary Figure S11. Proposed signaling pathways for ISC fate decision and proliferation. CCN1 binds to integrins $\alpha_v\beta_3/\alpha_v\beta_5$ on, presumably, Paneth cells and *Lgr5*+ ISCs, which elicits two distinct signaling pathways bifurcating downstream of FAK. integrin-FAK signaling results in NF-κB activation and subsequent induction of Jag1, a Notch ligand, which may activate Notch with a reduction of MFNG in a Wnt high environment. Notch signaling is important for ISC fate decision in the crypt base. For the regulation of Wnt signaling, FAK recruits and activates Src, leading to YAP stabilization and activation through phosphorylation at tyrosine 357. Active YAP promotes the expression of Wnt antagonist *Dkk1*, thus reducing Wnt signaling important for ISC proliferation. Concurrently, YAP can further reinforce the expression of *Ccn1* in the crypt, suggesting a regulatory loop between CCN1 and YAP.

Supplementary Table 1. List of antibodies

Antibodies	Source	Identifier	dilution		Figure number
			WB	IF or IHC	
Sheep anti-Cyr61/CCN1	R&D Systems	AF4055, Lot CALW0114051	1:800		Fig.1d, Fig.S5a
Rabbit anti-CCN1	homemade			1:100	Fig.1c, Fig.7a, Fig.S1b, Fig.S9c
Mouse anti-integrin alpha v	Chemicon	MAB 1953		1:200	Fig. S1a
Mouse anti-Sucrase-Isomaltase (C-8)	Santa Cruz	sc-393470, K2717	1:1000		Fig.2d
Mouse anti-Na^{+}/K^{+}-ATPase β1 (E-4)	Santa Cruz	sc-376406, A2918	1:1000		Fig.2d
Rabbit anti-activated Notch1 (N1-ICD)	abcam	ab8925, Lot GR3269530-11	1:500	1:100	Fig.4a,b, Fig5c, Fig.7e, Fig.S10
Rabbit anti-activated Notch1 (N1-ICD)	abcam	Ab52301		1:50	Fig. S4b
Mouse anti-Hes1 (A12)	Santa Cruz	Sc-166378		1:50	Fig. S4a
Rabbit anti-Notch 2 (25-255)	Santa Cruz	sc-5545, Lot L1812	1:500		Fig.4b
Rabbit anti-Jagged-1	abcam	ab7771	1:500		Fig.4b, Fig5c, Fig.7e
Goat anti-Dll-4	R&D Systems	AF1389, Lot: JP011503A	1:500		Fig.4b
Goat anti-Dll-1	abcam	ab85346, Lot: GR176201-33	1:500		Fig.4b
Rabbit anti-β-Catenin	Cell Signal Technology	Cat.# 9562 Lot:3	1:1000		Fig.4e, Fig.6c,f, Fig.7e
Rabbit anti-Non-phospho (Active) β-Catenin (Ser45)	Cell Signal Technology	Cat.# 19807 Lot:3	1:1000	1:3000	Fig.4d,e, Fig.6c,f, Fig.7e
Rabbit anti-Sox9	EMD Millipore	AB5535		1:500	Fig. S4b
Rabbit anti-axin2	abcam	Ab307613, Lot: GR185092-16		1:50	Fig. S4a
Rabbit anti-Olfm4 (D6Y5A) XP® (Mouse Specific)	Cell Signal Technology	Cat.# 39141, Lot:1		1:400	Fig.3a, Fig.S2, Fig.S9c
Rabbit anti-Phospho-YAP (Ser127)	Cell Signal Technology	Cat.# 4911, Lot: 5	1:1000		Fig.6c, Fig.S7b
Rabbit anti-Phospho-NF-κB p65 (Ser536)	Cell Signal Technology	Cat.# 3031 Lot: 11	1:500	1:100	Fig5c, Fig.S3b
Mouse anti-NF-κB p65	Cell Signal Technology	Cat.# 6956, Lot: 9	1:1000		Fig5c, Fig.S3b
Rabbit anti-YAP	Cell Signal Technology	Cat.# 4912 Lot:1	1:500	1:100	Fig.6c, Fig.7a, Fig.7e, Fig.S7a, Fig.S9a
Rabbit anti-E-Cadherin (24E10)	Cell Signal Technology	Cat.# 3195 Lot:2		1:200	Fig.1a, Fig.S1b
Rabbit anti-YAP1 (phospho Y357)	abcam	ab62751, Lot GRC3220921-1	1:1000		Fig.6c, Fig.S7b
Rabbit anti-Lysozyme	DAKO	A0099, Lot:20025086		1:1000	Fig.3e,g, Fig.5a,e,f, Fig.6a, Fig.7a,c, Fig.S5b, Fig.S6b, Fig.S9c, Fig.S10
Rabbit anti-Mucin 2 (H-300)	Santa Cruz	sc-15334 Lot:K0315		1:500	Fig.3e,g, Fig.5a,e,f, Fig6a, Fig.7a,c, Fig.S5b, Fig.S6b, Fig.S9c, Fig.S10
Rabbit anti-Chromogranin A	Proteintech	10529-I-AP		1:500	Fig.3e Fig.S5b
Rabbit anti-MFNG	Bioss Antibodies	Bs-12390R		1:200	Fig.S3a, Fig.S10
Chicken anti-GFP	abcam	ab13970		1:1500	Fig.1a, Fig.5a, Fig.6a,h, Fig.7c,f, Fig.S1a, Fig.S5b
Rabbit anti-Ki67 [SP6]	abcam	ab16667		1:250	Fig.3a, Fig.5a, Fig.6a,h, Fig.7c,f, Fig.S5b
Rabbit anti-Src (184Q20)	ThermoFisher Scientific	AHO1152 Lot:TF272605	1:500		Fig.6c
Rabbti anti-phospho-Src (Tyr419)	ThermoFisher Scientific	44-660G Lot:2196854	1:1000		Fig.6c
Rabbit anti-Lats1 (C66B5)	Cell Signal Technology	Cat #3477	1:500		Fig.6c
Rabbit Phospho-LATS1 (Thr1079) (D57D3)	Cell Signal Technology	Cat #8654	1:500		Fig.6c
Rabbit anti-GAPDH	Cell Signal Technology	2118, Lot 2	1:5000		Fig.1d, Fig.2d, Fig.S5a
Mouse anti-β-actin	abcam	ab8226	1:20000		Fig.4b,e, Fig. 5c, Fig.6c,f, Fig.7e, Fig.S3a,b, Fig.S7b
IRDye 800CW–conjugated goat anti-rabbit IgG	LI-COR	925-32211	1:20000		
IRDye 800CW–conjugated goat anti-goat IgG	LI-COR	925-32214	1:20000		
IRDye 680RD–conjugated goat anti-rabbit IgG	LI-COR	925-68071	1:20000		
IRDye 680LT–conjugated goat anti-mouse IgG	LI-COR	925-68020	1:20000		
Alexa Fluor 546 Goat anti-Rabbit IgG	Invitrogen	A11035 Lat:2040292		1:500	
Alexa Fluor 488 Goat anti-Chicken IgG	Invitrogen	A11039 Lat:109413		1:500	
Alexa Fluor 680 Goat anti-Mouse IgG	Invitrogen	A21058		1:500	
APC Goat anti-Rabbit IgG	Invitrogen	A10931		1:500	

Supplementary Table 2. Primers

Gene	Forward	Reverse
Axin2	GAGATGACGCCTGTGGAACC	CCTGCTCAGACCCCTCCTTT
Atoh1	ACA GAA GTG ACG GAG AGT TTT C	AGG GTG CAG GGA TAT TTG TC
CD44	CCACAGCCTCCTTTCAATAACC	GGAGTCTTCGCTTGGGGTA
Ccn1	AAA GGC AGC TCA CTG AAG	GCC GGT ATT TCT TGA CAC
CypE	TCC ACA AAC CAC AAT GGC ACA GGG	TGC CGT CCA GCC AAT CTG TCT TAT
Dkk-1	CGCTGCATGAGGCACGCTAT	GGCGGCGTTGTGGTCATTAC
Dkk-2	ATGATGGAAACCTGGATTGGAA	GAGGCACATAACGGAAGCA
sFRP-5	GGGGACCGAAAGTTGATT	GCCCGTCAGGTTGTCTA
Wnt3a	GGT GGC TTT GTC CAG AAC AG	GTG GCT GAG GGT GTC AAA G
Wnt5a	ACACAACAATGAAGCAGGCCGTAG	GGAGTTGAAGCGGCTGTTGACC
Wnt5b	CTGCTGACTGACGCCAACT	CCTGATACAACTGACACAGCTTT
Wnt9b	AAG TAC AGC ACC AAG TTC CTC AGC	GAA CAG CAC AGG AGC CTG ACA C
Ephb2	GCC GTG GAA GAA ACC CTG AT	GTT CAT GTT CTC GTC GTA GCC
Myc	CTC AGT GGT CTT TCC CTA CCC G	TGT CCA ACT TGG CCC TCT TGG C
Jag-1	CAG TGC CTC TGT GAG ACC AA	AGG GGT CAG AGA GAC AAG CA
Dll1	TAC ACA TGT TCC TGC CGA CC	AGG TGC AAG AGA AGC TGT CC
Dll4	GGT CGC CTG TGC AAT GAA TG	TTC TTG CAC GGA GAG TGG TG
Hes1	AGA GGC TGC CAA GGT TTT TG	TCC CAC TGT TGC TGG TGT AGA
Hey1	GTT AAC TCC TCC TTG CCC GC	TCG TTG GGG ACA TGG AAC AC
HeyL	GTC TTG CAG ATG ACC GTG GA	CTC GGG CAT CAA AGA ACC CT
YAP	ACC AAT AGT TCC GAT CCC TTT C	TGT CTC CTG TAT CCA TTT CAT CC
Lgr5	CAC CCC AAT GCG TTT TCT	GAT GGT ATC AGG CTC TGT AAG G
Olfm4	GCC ACT TTC CAA TTT CAC	GAG CCT CTT CTC ATA CAC
Ascl2	CCT CTC TCG GAC CCT CTC TCA G	CAG TCA AGG TGT GCT TCC ATG C
Sox9	CTG GAG GCT GCT GAA CGA GAG	CGG CGG ACC CTG AGA TTG C
Atp1a1	GTA TCG GAG CAT GGT GAC AAA	TCG TCC ATA GAC ACT TCC TTC T
Atp1b1	TCG GGA CCA TCC AAG TAA TGC	GGG GAA TCT GTG TCA ATC CTG
Slc2a1	GGT ATC AAT GCT GTG TTC TAC TAC TCA A	CCA CAG TGA AGG CCG TGT T
Slc2a2	GGG ACA AAC TTG GAA GGA TCA A	AAA TTT GGA ACA TCC CAT CAA GAG
Slc5a1	TGG TGT ACG GAT CAG GTC ATT G	TTC AGA TAG CCA CAC AGG GTA CAG
Alpi	CCA CAA GGC TTC TAC CTC TTT	TTA GCG TGT CCT TCT CAT TGG
ChgA	AGC ATC CAG TTC CCA CTT	CTC TGT CTT TCC ATC TCC ATC C
Lyz1	CAA AGA GGG TGG TGA GAG ATC	TGA GAA AGA GAC AGA ATG GGC
Muc2	TTT CAA GCA CCC CTG TAA CC	AGG TCC TGG TGT TGA ACC TG
Claudin1	TGG GGC TGA TCG CAA TCT TT	ATG GGG GTC AAG GGG TCA TA
Claudin2	CAG TGT CTC TGG CAA GCT GA	CTC TTC GAA AGG ACG GCT CC
Claudin3	CCT AGG AAC TGT CCA AGC CG	CCC GTT TCA TGG TTT GCC TG
Claudin7	CAA GGG CCC GCA TAC TTT CT	TGG TTC CAG ACA AAA GCG GT
Claudin8	GGA ATG CCA ATC CAT CAC GC	CTC TTT TAT CCC CAG GCC CC
Zo-1	GAG CAG GCT TTG GAG GAG AC	TGG GAC AAA AGT CCG GGA AG
Zo-2	AGC TTG TAG TTC TGA GCC GC	CCG ACA CGG CAA TTC CAA AT
Zo-3	ATT GTT TCC AGG CCC CTC CT	AGA GAC AGC TAT GAA GC
Ccn1 (-115b) for ChIP	AAAAGGTGCAACGGAGCCAG	GTGTTGCAGTGACGTAGCTC
Ccn1 (-2.1kb) For ChIP	TCTCCGAGATTGCCCAGTTG	CGTTCAAACAGACACGCCAT
