# Supplementary material for: CCN1 interacts with integrins to regulate intestinal stem cell proliferation and differentiation
Source: Nat Commun. 2022 Jun 3;13:3117. doi: 10.1038/s41467-022-30851-1 (PMC9166801; doi:10.1038/s41467-022-30851-1)
Supplement: Supplementary file 3 — Reporting Summary [file 41467_2022_30851_MOESM3_ESM.pdf]

nature research

Corresponding author(s): Joon-Il Jun

Last updated by author(s): May 14, 2022

Reporting Summary

Nature Research wishes to improve the reproducibility of the work that we publish. This form provides structure for consistency and transparency in reporting. For further information on Nature Research policies, see Authors & Referees and the Editorial Policy Checklist.

Statistics

For all statistical analyses, confirm that the following items are present in the figure legend, table legend, main text, or Methods section.

n/a	Confirmed	
☐	☒	The exact sample size (*n*) for each experimental group/condition, given as a discrete number and unit of measurement
☐	☒	A statement on whether measurements were taken from distinct samples or whether the same sample was measured repeatedly
☐	☒	The statistical test(s) used AND whether they are one- or two-sided<br>*Only common tests should be described solely by name; describe more complex techniques in the Methods section.*
☒	☐	A description of all covariates tested
☒	☐	A description of any assumptions or corrections, such as tests of normality and adjustment for multiple comparisons
☐	☒	A full description of the statistical parameters including central tendency (e.g. means) or other basic estimates (e.g. regression coefficient) AND variation (e.g. standard deviation) or associated estimates of uncertainty (e.g. confidence intervals)
☐	☒	For null hypothesis testing, the test statistic (e.g. *F*, *t*, *r*) with confidence intervals, effect sizes, degrees of freedom and *P* value noted<br>*Give P values as exact values whenever suitable.*
☒	☐	For Bayesian analysis, information on the choice of priors and Markov chain Monte Carlo settings
☒	☐	For hierarchical and complex designs, identification of the appropriate level for tests and full reporting of outcomes
☒	☐	Estimates of effect sizes (e.g. Cohen's *d*, Pearson's *r*), indicating how they were calculated

Our web collection on statistics for biologists contains articles on many of the points above.

Software and code

Policy information about availability of computer code

Data collection	For microscopic images, Image Pro Insight V.8.0 (Media Cybernetics), ZEN Black version 8.1.0.484 (Zeiss), Leica LAS X Software Version 5.0.3 (Leica). For immunoblot analysis, Image Studio™ Software V.5.0 (LI-COR). For Fluorescence plate reading, Victor3 WorkOut™ version 1.5 (PerkinElmer). For qPCR analysis, CFX Manager version 2.1 (Bio-Rad). For oligonucleotide probe design of Ccn1 for in situ hybridization, OligoMinerApp version 1.0.4 (http://oligominerapp.org).
Data analysis	GraphPad Prism9 software version 9.3.1 (Dotmatics), Origin pro 2020 (64-bit) SR1, V.9.7.0.188 (OrginLab Corp.), Adobe Illustrator/ Photoshop CC2 021 (Adobe), and ImageJ bundled with 64-bit Java 1.8.0_172 (NIH).

For manuscripts utilizing custom algorithms or software that are central to the research but not yet described in published literature, software must be made available to editors/reviewers. We strongly encourage code deposition in a community repository (e.g. GitHub). See the Nature Research guidelines for submitting code & software for further information.

Data

Policy information about availability of data

All manuscripts must include a data availability statement. This statement should provide the following information, where applicable:

- Accession codes, unique identifiers, or web links for publicly available datasets
- A list of figures that have associated raw data
- A description of any restrictions on data availability

Data supporting the findings of this work are available within the paper and the Supplementary Information files. Source data for all tabulated Figures and all exact p-values are provided with this paper in Source Data file.

Field-specific reporting

Please select the one below that is the best fit for your research. If you are not sure, read the appropriate sections before making your selection.

☒ Life sciences ☐ Behavioural & social sciences ☐ Ecological, evolutionary & environmental sciences

For a reference copy of the document with all sections, see nature.com/documents/nr-reporting-summary-flat.pdf

Life sciences study design

All studies must disclose on these points even when the disclosure is negative.

Sample size

No statistical methods were used to predetermine the sample size in the study. Sample sizes were chosen according to established practice in the laboratory (Jun and Lau, 2015, Jun and Lau, 2020). For western blotting, and immunohistochemistry, samples were derived from at least three animals or 3 independent culture of intestinal organoids per genotype. For RT-PCR experiments, total RNAs were pooled from 4 mice, each sample was assayed in quadruplicates. For mice body weight comparison, 5 mice per each group were evaluated at the indicated time point. For in vivo paracellular permeability assay and measurement of glucose concentrations, 3 mice per each group were used. For ChIP analysis, intestinal crypts were pooled from 3 mice per group.

Data exclusions

No data point has been excluded.

Replication

CCN1 proteins were obtained from a commercial source (R&D system) with distinct cellular origins and different purification methods and tested for the activities. These experiments confirmed that the results could be reproduced with commercially available CCN1 proteins. All important assays and experiments were repeated at least three times and all replications were successful. The exact number of replicates for each experiment is described in figure legends.

Randomization

For mice study, we used both male and female mice only based on age and weight and most mice were from different cages to suffice the required numbers due to the maintenance of being heterozygous for Cre allele. For the analysis of the crypt and villus length, the intestinal tissues (duodenum, jejunum, and ileum) were stained with H&E and the images were taken from randomly selected fields. Approximately 3 to 4 crypts/villi per field were analyzed.

Blinding

We mostly attempted to randomize to measure and evaluate the phenotypes in mice upon Ccn1 deletion by tamoxifen treatment. However, Lgr5-EGFP-IRES-creERT2 mice exhibit clonally mosaic expression of Cre recombinase. Therefore, we only chose the crypts that express GFP, the surrogate for Lgr5 and Cre, when we analyzed the expression of certain differentiation markers and markers for Notch activation or Wnt activation in immunofluorescence staining.

Reporting for specific materials, systems and methods

We require information from authors about some types of materials, experimental systems and methods used in many studies. Here, indicate whether each material, system or method listed is relevant to your study. If you are not sure if a list item applies to your research, read the appropriate section before selecting a response.

Materials & experimental systems

n/a	Involved in the study	
☐	☒	Antibodies
☐	☒	Eukaryotic cell lines
☒	☐	Palaeontology
☐	☒	Animals and other organisms
☒	☐	Human research participants
☒	☐	Clinical data

Methods

n/a	Involved in the study	
☒	☐	ChIP-seq
☒	☐	Flow cytometry
☒	☐	MRI-based neuroimaging

Antibodies

Antibodies used

anti-CCN1 Ab (from house; 1:400 for IF), Sheep anti-Cyr61/CCN1 (R&D Systems AF4055; 1:800 for WB), anti-integrin av (Chemicon; 1:200 dilution for IF), anti-Sucrase-Isomaltase (Santa Cruz sc-393470; 1:1000 dilution for WB), anti-Na+/K+-ATPase β1 (Santa Cruz sc-376406; 1:1000 for WB), anti-activated Notch1 (NICD)(abcam ab8925; 1:500 for WB, 1:100 for IF/abcam ab52301; 1:50 for IF), anti-Notch 2 (Santa Cruz sc-5545; 1:500 for WB), anti-Hes1 (Santa Cruz sb166378; 1:50 for IF), anti-Jagged-1 (abcam ab7771; 1:500 for WB), anti-Dll-4 (R&D Systems AF1389, 1:500 for WB), anti-Dll-1 (abcam ab85346; 1:500 for WB), anti-β-Catenin (Cell Signal Technology # 9562; 1:1000 for WB), anti-Non-phospho (Active) β-Catenin (Ser45)(Cell Signal Technology # 19807; 1:1000 for WB, 1:3000 for IF), anti-Sox9 (EMD Millipore AB5535; 1:500 for IF), anti-Axin2 (abcam ab307613; 1:50 for IF), anti-Olfm4 (D6Y5A)(Cell Signal Technology # 39141; 1:400 for IF), anti-Phospho-Yap (Ser127)(Cell Signal Technology # 4911; 1:1000 for WB), anti-Phospho-NF-κB p65 (Ser536)(Cell Signal Technology # 3031; 1:500 for WB, 1:100 for IF), anti-NF-κB p65 (Cell Signal Technology # 6956; 1:1000 for WB), anti-Yap (Cell Signal Technology # 4192; 1:500 for WB, 1:100 for IF/IHC, and 2 ug Ab per 25 ug lysates for chromatin immunoprecipitation), anti-E-Cadherin (24E10)(Cell Signal Technology # 3195; 1:200 for IF), anti-Yap1 (Y357)(abcam ab62751; 1:1000 for WB), anti-Lysozyme (DAKO A0099; 1:1000 for IF), anti-Mucin 2

(H-300)(Santa Cruz sc-15334; 1:500 for IF), anti-Chromogranin A (Proteintech 10529-I-AP; 1:500 for IF), anti-MFNG (Bioss Antibodies Bs-12390R; 1:200 for IF), anti-GFP (abcam ab13970; 1:1500 for IF), anti-Ki67 [SP6](abcam ab16667; 1:250 for IF), anti-Src (184Q20)(ThermoFisher Scientific AHO1152; 1:500 for WB), anti-phospho-Src (Tyr419)(ThermoFisher Scientific 44-660G; 1:1000 for WB), anti-Lats1 (C66B5)(Cell Signal Technology #3477; 1:500 for WB), anti-Phospho-LATS1 (Thr1079) (D57D3)(Cell Signal Technology #8654; 1:500 for WB), anti-GAPDH (Cell Signal Technology #2118; 1:5000 for WB), anti-β-actin (abcam ab8226; 1:20000 for WB), IRDye 800CW–conjugated goat anti-rabbit (LI-COR 925-32211; 1:20000 for WB), IRDye 800CW–conjugated goat anti-goat (LI-COR 925-32214; 1:20000 for WB), IRDye 680RD–conjugated goat anti-rabbit (LI-COR 925-68071; 1:20000 for WB), IRDye 680LT–conjugated goat anti-mouse (LI-COR 925-68020; 1:20000 for WB), Alexa Fluor 546 Goat anti-Rabbit (Invitrogen A11035; 1:500 for IF), Alexa Fluor 488 Goat anti-Chicken (Invitrogen A11039; 1:500 for IF), Alexa Fluor 680 Goat anti-mouse (Invitrogen A21058; 1:500 for IF), and APC Goat anti-Rabbit (Invitrogen A10931; 1:500 for IF).

Validation

The home-made anti-CCN1 antibody was validated in Western blot analysis and immunohistochemistry in our previous publications (Jun and Lau, 2015, Jun and Lau, 2020, etc). All the commercially available antibodies have validation images on the supplier website (refer to catalog number for different suppliers).

Eukaryotic cell lines

Policy information about cell lines

Cell line source(s)

Sf9 insect cell line was obtained from American Type Culture Collection (CRL-1711™).

Authentication

For sf9 insect cells, morphology and ability of producing recombinant CCN1 proteins after baculovirus infection in the conditioned media were used for authentication.

Mycoplasma contamination

Sf9 insect cell line was tested negative for Mycoplasma contamination using commercial kit.

Commonly misidentified lines
(See ICLAC register)

Name any commonly misidentified cell lines used in the study and provide a rationale for their use.

Animals and other organisms

Policy information about studies involving animals; ARRIVE guidelines recommended for reporting animal research

Laboratory animals

Transgenic CCN1(CYR61)-EGFP (Ccn1EGFP/+) mice in FVB/N-Swiss Webster background were obtained from the Mutant Mouse Regional Resource Centers. Ccn1D125A/D125A and Ccn1DM/DM knock-in mice in a svJ129-C57BL/6J mixed background were back-crossed to the C57BL/6J strain 6 and 11 times, both male and female, 10-12 weeks of age with similar body weight (25-28 g). Ccn1D125A/D125A and Ccn1DM/DM knock-in mice and Ccn1flox/flox mice were generated in the lab (Monzon et al. 2017, Method Mol Biol). Ccn1ΔLgr5 mice were generated by crossing Ccn1flox/flox with Lgr5+-specific Cre deleter stain, Lgr5-EGFP-IRES-creERT2 (08875, Jackson Laboratory); YapΔLgr5 mice were generated by crossing Yapflox/flox (kindly provided by Dr. Duojia Pan) with Lgr5+-specific Cre deleter stain, Lgr5-EGFP-IRES-creERT2 (08875, Jackson Laboratory). All mice were housed in sterile static micro-isolator cages on autoclaved corncob bedding with water bottles. Both irradiated food and autoclaved water were provided ad libitum. The standard photoperiod was 14 hours of light and 10 hours of darkness. The housing facility maintained the temperature at 75 degree F and the relative humidity within the range of 40-60%.

Wild animals

The study did not involve Wild animals.

Field-collected samples

The study did not involve samples collected in the field.

Ethics oversight

Animal protocols (ACC#20-178) were approved by the Institutional Animal Care and Use Committee of The University of Illinois at Chicago

Note that full information on the approval of the study protocol must also be provided in the manuscript.
